# ARAN: Age-Restricted Anonymized Dataset of Children Images and Body Measurements

**DOI:** 10.3390/jimaging11050142

**Published:** 2025-04-30

**Authors:** Hezha H. MohammedKhan, Cascha Van Wanrooij, Eric O. Postma, Çiçek Güven, Marleen Balvert, Heersh Raof Saeed, Chenar Omer Ali Al Jaf

**Affiliations:** 1 Zero Hunger Lab, Department of Econometrics & Operations Research, Tilburg School of Economics and Management, Tilburg University, 5037 AB Tilburg, The Netherlands; c.vanwanrooij@tilburguniversity.edu (C.V.W.); m.balvert@tilburguniversity.edu (M.B.); 2 Department of Cognitive Science and Artificial Intelligence, Tilburg School of Humanities and Digital Sciences, Tilburg University, 5037 AB Tilburg, The Netherlands; e.o.postma@tilburguniversity.edu (E.O.P.); c.guven@tilburguniversity.edu (Ç.G.); 3 College of Medicine, University of Sulaimani, Kurdistan Region, Sulaymaniyah 46002, Iraq; heersh.raof@univsul.edu.krd (H.R.S.); chenar.jaff@univsul.edu.krd (C.O.A.A.J.)

**Keywords:** image-based body shape estimation, convolutional neural networks, dataset

## Abstract

Precisely estimating a child’s body measurements and weight from a single image is useful in pediatrics for monitoring growth and detecting early signs of malnutrition. The development of estimation models for this task is hampered by the unavailability of a labeled image dataset to support supervised learning. This paper introduces the “Age-Restricted Anonymized” (ARAN) dataset, the first labeled image dataset of children with body measurements approved by an ethics committee under the European General Data Protection Regulation guidelines. The ARAN dataset consists of images of 512 children aged 16 to 98 months, each captured from four different viewpoints, i.e., 2048 images in total. The dataset is anonymized manually on the spot through a face mask and includes each child’s height, weight, age, waist circumference, and head circumference measurements. The dataset is a solid foundation for developing prediction models for various tasks related to these measurements; it addresses the gap in computer vision tasks related to body measurements as it is significantly larger than any other comparable dataset of children, along with diverse viewpoints. To create a suitable reference, we trained state-of-the-art deep learning algorithms on the ARAN dataset to predict body measurements from the images. The best results are obtained by a DenseNet121 model achieving competitive estimates for the body measurements, outperforming state-of-the-art results on similar tasks. The ARAN dataset is developed as part of a collaboration to create a mobile app to measure children’s growth and detect early signs of malnutrition, contributing to the United Nations Sustainable Development Goals.

## 1. Introduction

In recent years, there have been advancements in applying deep learning to infer anthropometric measurements from 2D images in different contexts. For instance, ref. [1] estimated the Body Mass Index (BMI), a common measure related to the height and weight of a person, directly from facial images. Other applications are the estimation of body measurements from 2D images for the virtual fitting of clothing [2], the generation of 3D body-shape meshes from 2D images [3], and for evaluating nutritional status [4]. A significant hurdle in the development of algorithms is the relative scarcity of high-quality domain-specific datasets. Much of current research is based on data collected on the Internet [5], often lacking reliable ground-truth labels. With this paper, we offer a dataset whose ground-truth labels are collected by medical experts in a hospital setting. It is essential to prioritize efforts to collect and curate datasets with precise and accurate annotations, ensuring their inclusiveness and comprehensiveness for the intended applications, to advance further and develop reliable AI solutions for pressing problems. Child growth and development are critical areas where the lack of real-world image data has been particularly pronounced. The World Health Organization (WHO) defines the nutritional status of children as a critical global concern. According to recent statistics jointly published by UNICEF 148 million children under the age of five are stunted and 45 million are wasted, with an additional 37 million classified as overweight. Furthermore, 45% of global deaths in children under five years of age are related to nutrition [6]. Children are manually screened and measured for the detection of malnutrition, which is time-consuming, and early intervention is crucial, especially for children with unique medical conditions [7]. There have been attempts to automate this process and build intelligent systems to make it more efficient and less time-consuming; for example, one approach suggests monitoring child growth through hands X-rays [8], but WHO growth standards do not support this approach. Another approach is to predict body measurements and plot them by age [9,10], which is the recommended WHO growth monitoring standard. However, the lack of data in this domain is slowing progress compared to the urgent need to develop such a support tool.

**Figure 1:**
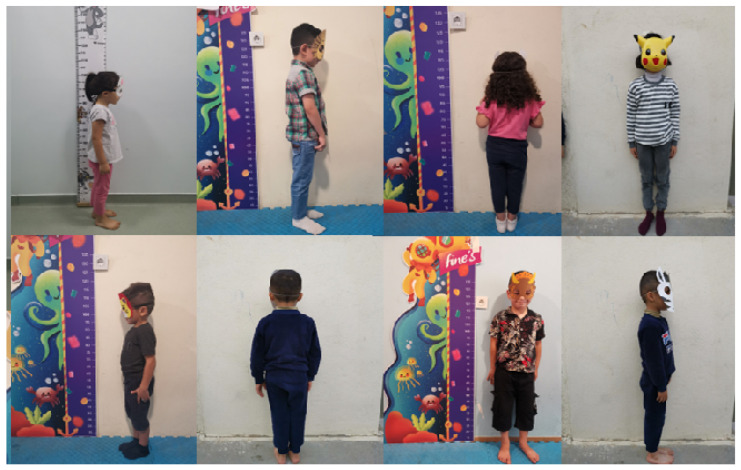
ARAN dataset examples from different viewpoints.

In this paper, we address this data scarcity challenge by presenting a novel dataset called the ARAN (Age-Restricted AnoNymized) dataset specifically tailored and intended for AI-based tasks related to monitoring the growth and development of children between the ages of 16 and 98 months. The ARAN dataset consists of anonymized full-body images of children captured from four different points of view, accompanied by the following four anthropometric measurements: stature (height), weight, head circumference, and waist circumference. Moreover, the anonymized nature of the dataset protects children’s privacy, enabling its safe and ethical utilization. Figure 1 shows eight examples of the images contained in the ARAN dataset. To the best of our knowledge, this is the first publicly available dataset featuring children’s body images with annotated body measurements that is compliant with the General Data Protection Regulations (GDPR). It has significant potential for applications such as malnutrition detection, garment fitting, and 3D body reconstruction.

A set of experiments was carried out that employ contemporary AI architectures to establish benchmark metrics for body measurements using the ARAN dataset. The experimental setup featured the use of prominent deep learning models, namely MobileNetV3 [11], ResNet50 [12], DenseNet121 [13], and Swin Transformer [14]. These experiments provide a foundational framework for researchers to improve the baseline performance outlined in our study, thereby leveraging the ARAN dataset for further advancements.

### 1.1. Related Work

#### 1.1.1. Human Body Image Datasets

Numerous datasets are available to investigate human body shapes and poses, encompassing both 3D body shapes and 2D images. These datasets serve as valuable resources for research and development purposes [15,16]. However, these datasets are not labeled with body measurements, which limits their use for the application of predicting body measurements from 2D images. The use of principal component analysis, as proposed in [17], enables the analysis of 3D body shapes to capture variations between different body shapes and poses. These variations are expressed in arbitrary units that can serve as ground truth for body scans; however, they need to be translated to be understood in metric units.

The available datasets are limited in their applications, as most existing ones primarily focus on adult populations, lacking a comprehensive representation of children. For example, the CAESAR dataset [18], which consists of 3D body scans of adult participants, is limited in scope as it only includes data on adult individuals and lacks information on children. Similarly, the MPII Human Pose dataset [19], the COCO dataset [20], and other commonly used datasets focus primarily on adult populations and do not provide body measurements annotations or images of children. There are also datasets of virtual body shapes, often associated with parameterized models like SMPL [21], which enable character animation and human body analysis. Hesse et al. [22] used a similar approach to create the SMIL model, the first of its kind for capturing 3D shape and motion in babies. However, this dataset was limited to 35 babies and relied on images of moving babies. To the best of our knowledge, the only two public datasets that contain data of children are the AGORA dataset [23] and Toddler Manikins [24], in addition to a restricted access dataset from a physiotherapist clinic in Brazil [25]. AGORA provides textured human scans in various poses and natural clothing and includes 257 child images including SMPL-X body model fittings. Toddler Manikins provides 18 3D models based on body shape data from 67 toddlers in seated positions. The body measurements for each 3D model are publicly available.

The development of the ARAN dataset is motivated by the scarcity of suitable image databases for young children with standardized shapes and postures coupled with anthropometric measurements. The ARAN dataset has three advantages: (1) standardized poses, (2) 512 children (more than AGORA), and (3) four viewpoints per child.

#### 1.1.2. Predicting Body Measurements

The field of predicting body measurements from 2D images has evolved significantly, employing various methods [1,5,9,22]. In this section, we review several of the CNN-based architectures, highlighting their limitations and performances. Pre-trained CNN architectures are widely utilized in image analysis through transfer learning. Among these, the MobileNet family [11] stands out for its exceptional balance between speed and performance [26], making it highly suitable for real-time applications—a key consideration for our research. On the other hand, more complex architectures such as DenseNet [13], ResNet [12,25], and Inception [27], while computationally intensive, offer advanced feature extraction capabilities. These models are particularly well-suited for our tasks due to the small size of our dataset and the necessity for robust feature extraction.

In addition to the pre-trained models mentioned, customized CNN architectures have also been developed. Ref. [28] introduced a part-based shape model, and [29] proposed a skeleton-driven annotation technique for body measurements that outperformed methods like HKS-Net [30], HMR [31], and SMPLify [32]. Both papers achieve lower mean absolute errors (MAEs) for various body measurements and introduce new datasets as these customized CNNs require large and diverse datasets. However, none of these datasets include images of children or children’s body shapes. Despite these advances, a common challenge remains the lack of standardization and concerns about generalizability to children’s body shapes, and this is mainly due to the general data scarcity in this domain, and specifically the lack of children’s data, which serves as the main motivation of this paper.

### 1.2. Outline of the Paper

The remainder of this paper is organized as follows: In Section 2, details of our data collection procedure, sample population, and the characteristics of the finalized dataset are provided. In Section 3, we present the experiments that were carried out on the dataset and the results we obtained in Section 3.4. In Section 4, we discuss the outcomes of our paper, comparing both the dataset and experiment results with state-of-the-art literature. Finally, concluding the paper, we highlight key insights and future works in Section 5.

## 2. The ARAN Dataset

The ARAN dataset consists of images of children and their body measurements. In this section, we provide an overview of the data collection procedure, sample population, and the finalized data set.

### 2.1. Data Collection Procedure

**Figure 2:**
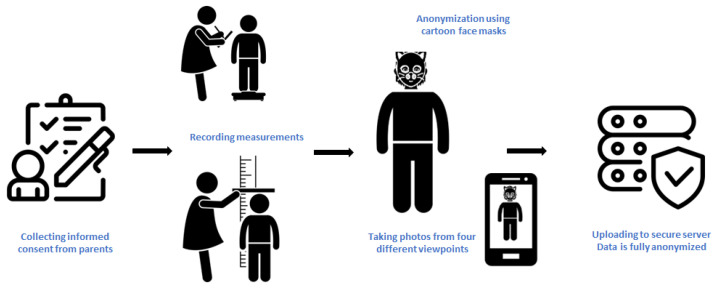
Schematic representation of the data collection procedure.

The data collection was conducted between July 2022 and April 2023, at the Anwar Sheikha Medical City and dr. Jamal Ahmed Rashid’s Paediatric Teaching Hospital in Sulaimaniyah, Kurdistan Region, Iraq. Upon the arrival of the children from the target population at the pediatric department of the hospitals, parents were asked whether our team could measure their children and take photos for our research. The conversation with the parents adhered to ethics committee guidelines, and comprehensive details regarding research procedures, data collection, processing, and storage were conveyed to parents to secure informed consent. After collecting informed consent from parents, the four body measurements were taken using the accurate measuring tools provided by the hospital, i.e., measuring tape and weighing scale. Anonymization as further explained in Section 2.3 was one of the biggest challenges of this research, as our data subjects are children. We considered face blurring as a means of anonymization, but future technologies may reverse the anonymization, e.g., through advanced hyperresolution algorithms. Hence, we decided that anonymization would be performed manually on the spot using face masks of cartoon characters to completely obscure the children’s faces. A unique identifier (child ID) was generated per child to label the photos and the corresponding measurements. Upon completing the data collection, the children were offered to keep the mask as a token of appreciation. Children who refused to collaborate also received the mask. Figure 2 provides a schematic illustration of the data collection procedure.

The four body measurements recorded were height in centimeters, weight in grams, and waist and head circumference in centimeters. We additionally recorded the children’s age in months and sex. Data collection adhered to the WHO directive on measuring children (https://www.who.int/tools/child-growth-standards, 20 March 2025). The nurses participating in the data collection were all pediatric nurses with a bachelor’s degree and had already been trained by the hospital to collect these measurements.

The image collection setup was designed to align with the daily environment of a healthcare center. The child was placed in a room with artificial white light in front of a wall. A measuring tape is visible in the background. Throughout the data collection procedure, the same mobile phone (Huawei Mate 20 Pro, F number f/1.8, exposure time 0.03) was used. All images were stored in an uncompressed JPG format with a resolution of 2736×3648 pixels and color profile sRGB IEC61966-2.1. To ensure consistency, nurses were instructed to take the photographs at a distance of one meter from the child and to hold the camera at a 90-degree angle from the ground, without tilting it forward or backward. The camera was held at the same height as the child’s face. Pictures were taken from four viewpoints: front, back, and both sides of the child.

#### Sample Population

**Figure 3:**
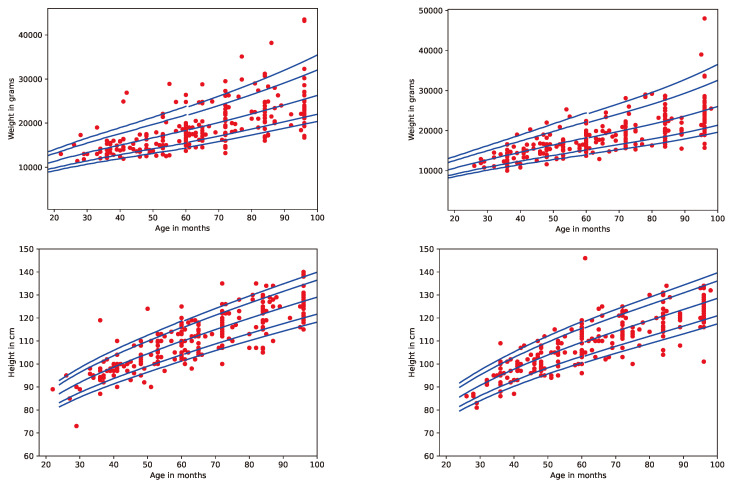
Growth charts for boys and girls in the ARAN dataset compared to WHO reference data. The blue lines represent the 3rd, 25th, 50th, 75th, and 97th percentiles, respectively. Data sources: WHO—Growth Reference, https://www.who.int/tools/growth-reference-data-for-5to19-years/indicators/, WHO—Growth Standard, https://www.who.int/tools/child-growth-standards/standards/, accessed on 20 March 2025.

The study population consisted of 512 children aged 16 to 98 months, i.e., 1.5 to 8 years, recruited from two hospitals in the Iraqi Kurdistan region. The sample included male and female children of varying socioeconomic backgrounds. All children were of Kurdish ethnicity. After running the campaign for 9 months, the dataset was compiled, with a balanced representation across age and sex groups Figure 3. Table 1 provides descriptive statistics of the dataset.

### 2.2. Dataset Contents

The ARAN dataset consists of images and body measurements of 512 children, with four views each resulting in a total of 2048 images. As illustrated in Figure 6, the dataset represents these images in four variants: (1) original, (2) center-cropped, (3) body-cropped, and (4) segmented.

An object-detection model, DETR ResNet-50 [33], was used to generate precise bounding boxes of the children’s bodies automatically. In the few cases where the automatic segmentation failed, we manually corrected the bounding box. For the center-cropped images, the bounding boxes were expanded by 5%. For the body-cropped images, no expansion was used.

**Figure 4:**
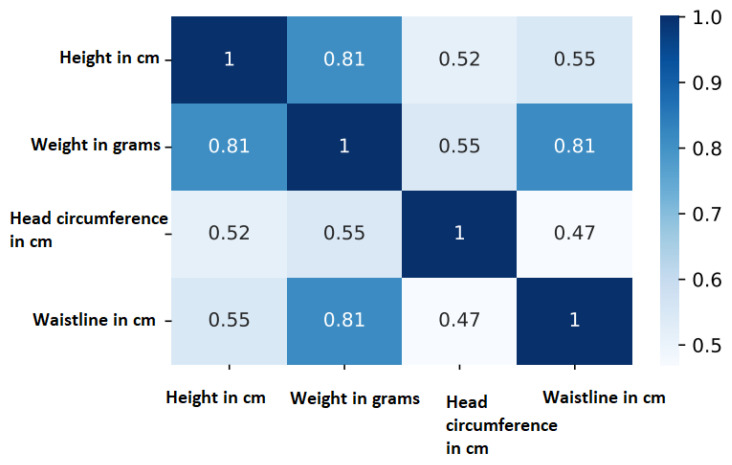
Pearson correlations between body measurements show that height and weight are strongly correlated in the ARAN dataset with the sample size of 512.

**Figure 5:**
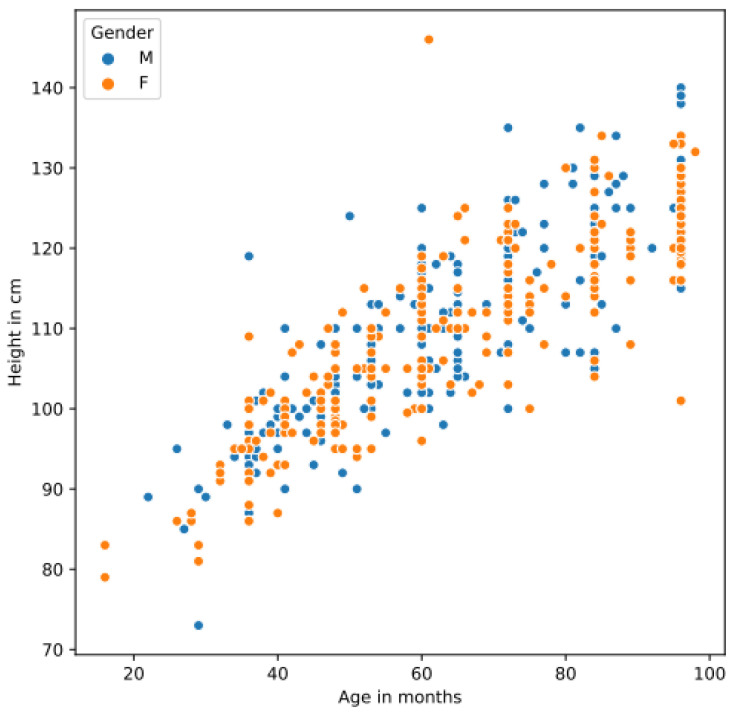
A scatter plot depicting the correlation between height and age in the ARAN dataset for both male and female children.

The four body measurements, the age in months, and the sex of each child were stored in an Excel file. Figure 4 shows that there are (obvious) correlations between body measurements and weight. Strong positive correlations exist between height and weight (0.81) and waistline and weight (0.81). Similarly, age and weight are correlated as illustrated for the male and female children in the ARAN dataset in Figure 5.

### 2.3. Challenges in Data Collection

The data collection process, while thorough, presented several challenges that required careful consideration and innovative solutions. One significant challenge was related to anonymization and complying with GDPR. Initially, the use of facial blurring was proposed as the anonymization method; however, this approach was rejected by the ethical committee due to the potential risks of reverse anonymization techniques that could be developed in the future. After multiple iterations, we developed the current anonymization technique involving face masks, which the ethics committee promptly approved. This method also addressed concerns regarding the visibility of identifiable features, such as birthmarks. Further, as per GDPR guidelines, there needs to be informed consent, so our data collection team had to spend considerable time explaining the science behind the concept and the overall research project. To make this procedure easier, there was an awareness campaign on the importance of measuring children under the age of five, and infographics were handed out to parents at the hospital. Another challenge was participant recruitment, as the data collection occurred in a hospital setting. Many of the children were present for medical reasons, often unwell, making it burdensome for them to participate in the study. This limited the number of participants we could involve and necessitated additional sensitivity in approaching potential participants and their families. Finally, clothing norms posed a methodological challenge. Due to the religious and cultural practices in the region where the study was conducted, it was not feasible to ask children to undress down to their undergarments, as is standard practice for accurate anthropometric measurements. Instead, we allowed parents to undress their children to the level they were comfortable with, which often meant the children remained fully dressed. While this approach ensured respect for cultural norms, it also made the body-shape estimation more difficult as clothing entirely covers the body surface and challenges feature preservation. As for face anonymization, while these masks entirely hide the face, this does not cause any loss of the necessary features, because we are motivated by the World Health Organization standards for detecting early signs of malnutrition, which mainly relies on body measurements.

## 3. Predicting Body Measurements

We use the ARAN dataset to train state-of-the-art deep learning algorithms on the regression task of predicting the four body measurements from the images. In what follows, we describe our methodology and experiments.

### 3.1. Pre-Processing

**Figure 6:**
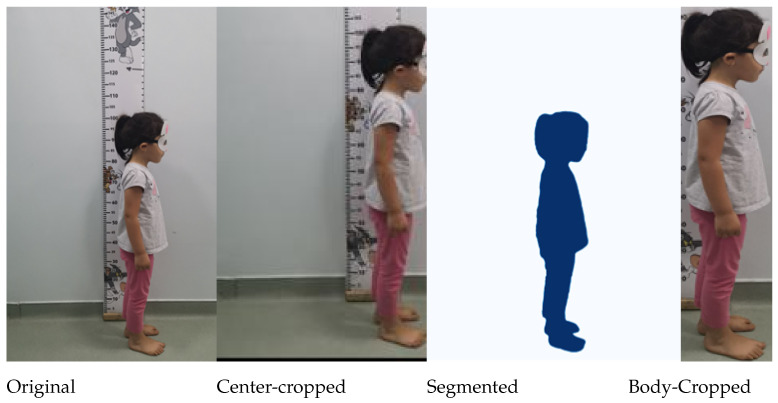
Variants of the dataset from left to right: original, center-cropped, body-cropped, and segmented.

From the four variants of the ARAN dataset shown in Figure 6, we used the center-cropped images for our experiments as we relied on pre-trained CNNs and Vision Transformers which accept center-cropped images for optimal results. To achieve uniform body-cropped image sizes, zero padding was applied to the top and bottom of each image. The amount of padding was determined based on the length of the longest bounding box (which requires no padding) and the shortest bounding box (which necessitates maximal padding). This padding procedure ensured that all images had equal dimensions. Consequently, all body-cropped images in the ARAN dataset have a standardized size of 1310×224×3.

### 3.2. Network Training

To effectively predict the body measurements from ARAN images, we selected a diverse set of state-of-the-art pre-trained models that have the best reported performances on a wide variety of tasks, i.e., CNNs and Vision Transformers. In our selection, we balanced efficiency, depth, and representation power. Each model was chosen for its unique strengths. MobileNetV3 (small CNN) [11] is optimized for low computational resource environments, ResNet50 [12] and DenseNet121 [13] are CNNs known for strong performance due to residual connections and dense connectivity. The Swin (Vision) Transformer (small) is a hierarchical model that captures both local and global features and the ViT-B/16 Vision Transformer [34] for high-level abstraction and fine-grained image analysis that traditional CNNs may miss. Due to the relatively small size of the ARAN dataset, all models were pre-trained on ImageNet, allowing them to leverage the visual features learned and effectively transfer knowledge to the task of human measurement prediction [35]. As is standard practice in transfer learning, the classifier heads of the models were removed and replaced by our regression head consisting of two dense layers. The output of the second dense layer passed through a linear output neuron.

To optimize the number of nodes in the dense layers, we experimented with various configurations, ranging from 20 to 100 nodes. Fewer nodes reduced performance, as the model could not learn intricate relationships in the data. Employing too many nodes led to overfitting. We found that an optimal number of nodes, i.e., 50, struck a balance between underfitting and overfitting, yielding the best performance on the regression task. Classifier parameters were held constant across models. Table 2 presents the total parameters for each model, including the projection layer. For all models, we set the learning rate to $3\times 10^{-5}$ and a batch size of 4.

During the first 10 epochs of training, the weights of the pre-trained network are frozen, while the weights of the weights of the regression head are unfrozen. Subsequently, all weights are unfrozen.

### 3.3. Evaluation Procedure

The trained models are evaluated using a standard regression metric, the Mean Absolute Error (MAE). Following the standard practices in this field, our results are reported as MAEs in terms of centimeters (cm) and kilograms (kg). To put our results into context, we evaluate the results obtained with a baseline model based on linear regression of age and sex. For each metric, we take the average of the three-fold cross-validation on the test set. In this approach, the data are split 80/20 train/test by randomly sampling three different splits based on the child ID (so samples are independent), stratified by age and sex (age split into 10 equally sized bins).

#### Linear Baseline

Given this correlation, we ran two sets of experiments: one experiment with known age, and another experiment without feeding age into the model and reflected on the results in Section 3.4.

To evaluate the significance of image features in our predictions, we established a baseline model that includes age and sex as predictors. This baseline allowed us to assess the additional contribution of image features in accurately estimating the body measurements, providing insight into how much image content enhances our predictive capabilities. By comparing the performance of the baseline model to that of the full models incorporating image features, we can determine the added value brought by the image content in achieving accurate measurement estimation. The linear baseline model is defined as:

(1) $$y_{i}=\beta_{0}+\beta_{1}{\mathrm{age}}_{i}+{\mathrm{sex}}_{i}+\epsilon_{i}$$

where $y_{i}$ is the target, e.g., height, and $\epsilon_{i}$ is the error term. Age is expressed in months and sex in dummy values: 0 = male and 1 = female. Since this is a simple model, we evaluated the full dataset using a cross-validation split of 70:15:15.

### 3.4. Results

The results of all the models on the test set are reported in Table 3. Observing the results, we see that all models outperform the baseline of Equation (1) listed in the top row. DenseNet121 emerged as the most effective model across all four measurements with the lowest MAE of 1.5 kg for weight, 2.54 cm for height, 2.53 cm for waist circumference, and 1.52 cm for head circumference.

The model’s performance for different metrics varies slightly. If we had to pick one model with consistent performance across all four measures, our preferred option would be the DenseNet121 model.

To further explore the impact of viewpoint on the prediction performance, we train DenseNet on each viewpoint independently to make a prediction, as well as on all four viewpoints to make an average prediction. The results are reported in Table 4. For the different viewpoints, the results suggest that the front viewpoint almost always gives the best prediction, and the back gives the worst prediction. As for the average prediction compared to one viewpoint, the average prediction from all viewpoints is always better compared to looking at it from one viewpoint.

## 4. Discussion

### 4.1. ARAN Dataset

The dataset presented in this paper holds great potential for studying and extracting body shape measurements from images. It is the first dataset of children’s bodies with body measurement annotations and can be used in various applications for detecting malnutrition, garments, and 3D reconstruction. Compared to existing datasets [5,15,18,23], the ARAN dataset, although the smallest in size, provides the largest number of images and annotations for small children and is the only dataset to provide free access to body measurements, as CAESAR is a commercial dataset. Table 5 provides a brief comparison between the ARAN dataset and other similar public and commercially available datasets. As can be seen, ARAN is unique in the age range by containing images of children’s bodies and associated body measurements.

The ARAN dataset has three main limitations. First, regarding the sample population, we only have 512 children with a total of 2048 images, which is considered small. Second, all participants are from one ethnic group and geographic area. Third, the children in our dataset are all wearing clothes that can add a burden to the prediction task and reduce the model’s performance.

### 4.2. Body Measurements

The initial experiments on this dataset employing pre-trained convolutional neural networks (CNNs) and Vision Transformers yielded interesting insights. We obtained promising results with an MAE of 1.51 kg for weight estimation and 2.54 cm for height estimation. The results of all six models exceeded the linear baseline in all four measurements. Among the six models, DenseNet121 offered the lowest MAE, which makes it the best performing for our dataset.

Our selection of CNNs and Vision Transformer architectures covered state-of-the-art models for visual tasks. The finding that DenseNet121 achieved the best performance indicates that its architecture is best suited for the task at hand. DenseNet121’s superior performance may be due to its dense connectivity, which improves feature reuse and gradient flow. DenseNet also displayed an efficient use of parameters, which leads to better generalization. These architectural characteristics make DenseNet121 particularly well suited for tasks requiring high accuracy, such as estimating body measurements. While ResNet50, ViT, and MobileNet are strong contenders, DenseNet121’s architecture strikes the proper balance between depth, feature richness, and computational efficiency for this particular image recognition task.

It is important to emphasize that these results fall short of meeting the stringent MAE criteria recommended by the SMART methodology manual for the practical detection of malnutrition. SMART Methodology stands for Standardized Monitoring and Assessment of Relief and Transitions. This methodology is designed to enhance the quality of nutrition and mortality surveys, particularly in humanitarian and development settings. It provides a consistent and reliable framework for collecting and analyzing survey data, ensuring that information is gathered using a single standardized approach. For a height prediction to be reliable for measuring children, an MAE of 1.4 cm is required. A measuring scale should not have an error of more than 50 g for children under two and 100 g for children over two years old. These are very ambitious results to aim for with the small datasets that are available for researchers, hence yielding the importance of the ARAN dataset. To meet the SMART requirements, it is important to expand the size of the dataset as these architectures require big data for training. But there is also more that can be achieved. Looking at the results reported in [10,36], MAEs of 0.9 cm and 1.2 cm, respectively, we see that standardized camera calibration and the absence of heavy clothing provide significantly better results in comparison to the results obtained from the ARAN dataset with less standardized camera calibration and full clothing of the children. We can also conclude from their experiments that the MAE is much smaller in [10] with more standardization regarding camera location, camera angle, the color of the body, and the standard human pose.

**Figure 7:**
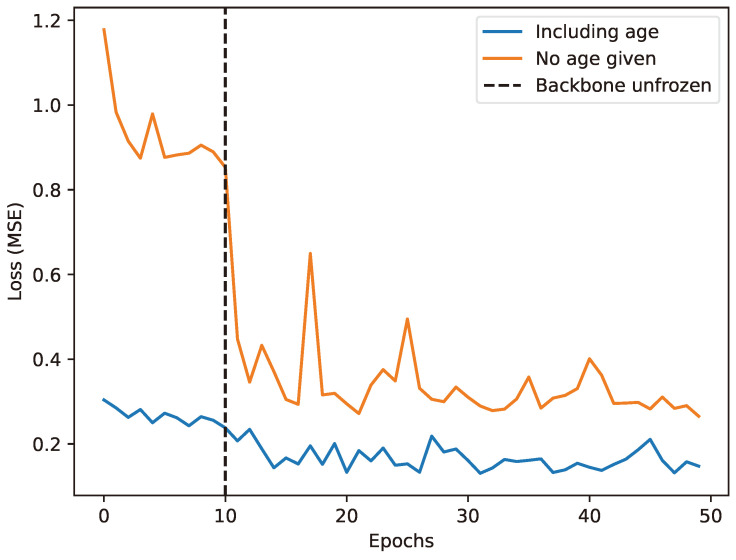
Test loss development during training when predicting height with age (blue line) or without age (orange line).

Given the established correlation between age and height, we examined the performances of the models with known and without age provided. The curve in Figure 7 shows the results of these two cases. We found that models trained with age as an input variable demonstrate faster convergence and better accuracies. This suggests that accounting for age-specific growth patterns can significantly enhance the accuracy of body measurement estimations and may help to meet the SMART requirements.

Potential biases in our dataset arise from the selective sample of children from a particular region. As illustrated in Figure 3, the measurements of our selective sample fit well with the WHO standard, although there are some outliers, i.e., measurements of children that are either obese or falling within the lower range of growth. To ensure the generalizability of our model to children from other regions, extending the dataset with additional images and measurements from children of other regions is required.

Furthermore, our investigation into viewpoints for all four measurements consistently produced superior MAE results from multiple viewpoints compared to considering measurements from a single viewpoint alone. This underscores the importance of multi-viewpoint data aggregation for more accurate assessments of body parameters.

## 5. Conclusions

Our study contributes significantly to building intelligent systems for child malnutrition detection through image analysis. We meticulously compile a dataset of 512 anonymized children in 2048 images, capturing their body profiles from four distinct viewpoints and including crucial body measurements: height, weight, head circumference, and waist circumference. By incorporating age and gender variables, we provide a comprehensive resource for the scientific community to study children’s physical development and facilitate early malnutrition detection through image analysis. We provide a simple yet effective method to overcome privacy and data protection concerns by wearing a face mask and anonymizing data before they are processed, making our approach safe and easy to replicate so that other researchers can collect more data and combine them with this dataset. Finally, we demonstrate how the dataset can be used with state-of-the-art architectures and models best suitable for this task and provide a benchmark so that researchers can build up on the accuracy of prediction, perhaps through image processing techniques and hyperparameter tuning. Future iterations of the ARAN dataset will be released, including more children with varying poses, so not just standing up in an A-shape but also sitting down and putting their hands up. In addition to providing an image embedding version of the dataset, which will be published on the website created to publish the ARAN dataset, every future iteration and addition to the dataset will be published on the website to be made available for the scientific community to use. In the future, research efforts should prioritize expanding the dataset size and enhancing diversity in samples by including children from different ethnic and racial backgrounds. This can be achieved by collecting data in multiple countries, which inevitably presents many challenges in terms of funding, obtaining ethical approval, and establishing multiple partnerships, or by collecting data in one country where the population is diverse and more representative of different races and ethnicities in the world. Future work should also explore alternative approaches to improve the prediction of body shape measurement estimations, which can be easier with larger datasets. Furthermore, we hope to see more work focused on the explainability and transparency of deep learning models in this line of research. As these support tools will be applied to children, ensuring transparency is important.

Our experimental results provide a foundational reference for subsequent research in this domain. Although initial findings suggest that AI models may not yet meet desired accuracy levels, they represent a crucial starting point for algorithm refinement. By providing both the dataset and baseline results, we aim to facilitate the development of more accurate and robust algorithms, supporting better assessments of malnutrition and effective interventions.

## Figures and Tables

**Table 1:** Descriptive statistics of body measurements in the ARAN dataset. The nutrition status of the children in the ARAN dataset is given in percentiles which indicate how a child’s growth compares to peers of the same age and sex: 25th percentile: Lower than 75% of peers; 50th percentile (median): Average—half of peers are above, half below; 75th percentile: Higher than 75% of peers.

	Height (cm)	Weight (g)	Head Circumference (cm)	Waistline (cm)	Age (Months)
count	512	512	512	512	512
mean	111.0	18,983	50.2	53.4	65.4
std	12.2	5184	2.5	5.4	20.2
min	73.0	9300	37.5	36.0	16.0
25%	101.0	15,200	49.0	50.0	48.0
50%	112.0	17,900	50.0	53.0	65.0
75%	120.0	22,000	52.0	55.0	84.0
max	146.0	48,000	69.0	84.0	98.0

**Table 2:** Parameters of Different Models.

Model	Total Param.	Classifier Param.
MobileNet V3 Small	961,109	5251
MobileNet V3 Large	3,025,253	5251
DenseNet121	7,010,357	5251
ResNet50	23,615,733	5251
Swin B	86,799,725	5251
ViT B_16	85,842,357	5251

**Table 3:** Comparison of the Mean Absolute Errors (MAEs) of the studied models on estimating the four body measurements: Height, Weight, Waist Circumference, and Head Circumference. The top row shows the MAEs of the baseline of Equation (1).

	MAE (cm)	MAE (kg)	MAE (cm)	MAE (cm)
Model	Height	Weight	Waist Circumference	Head Circumference
baseline	4.89	2.20	3.00	1.66
densenet121	**2.54**	**1.51**	**2.53**	**1.52**
mobilenet_v3_large	3.05	1.69	2.77	1.56
mobilenet_v3_small	3.21	1.78	2.75	1.61
resnet50	2.80	1.58	2.59	1.55
swin_b	3.16	1.78	2.77	1.57
vit_b_16	2.83	1.73	2.78	1.61

**Table 4:** Impact of viewpoint on the prediction of different metrics.

	MAE (cm)	MAE (kg)	MAE (cm)	MAE (cm)
Stance	Height	Weight	Waist Circumference	Head Circumference
front	2.56	1.59	**2.53**	1.67
left	2.63	1.78	2.75	1.63
back	3.02	1.69	2.87	1.57
right	2.86	1.58	2.63	1.53
all	**2.54**	**1.51**	**2.53**	**1.52**

**Table 5:** Comparison of Datasets commonly used for Body Measurement Prediction: Summary of Dataset Content, Characteristics, and Scope. As we can see, the ARAN dataset provides the largest amount of children compared to the existing datasets, along with important body measures that are required for measuring growth.

Dataset	Size	Num. Children	Type	Age Range	Annotations
MORPH-II	202k	0	Facial	16 to 77 years	BMI
CAESAR	11,808	0	Full Body	18 to 65 years	Age, Sex, BMI, 15 Body metrics
IMDB 23K	23K	0	Facial and Full Body	18+ years	BMI, Gender, BMI
AGORA	17K	257	Full Body	Not specified	None
**ARAN**	**2048**	**512**	**Full Body**	**16 to 98 months**	**Age, Sex, 4 body metrics**

## Data Availability

The Aran datasets and codes are made available for researchers on https://aranchilddata.uvt.nl/, 20 March 2025.
